# Classroom Seat Proximity Predicts Friendship Formation

**DOI:** 10.3389/fpsyg.2022.796002

**Published:** 2022-05-03

**Authors:** Sharon Faur, Brett Laursen

**Affiliations:** Department of Psychology, Florida Atlantic University, Boca Raton, FL, United States

**Keywords:** friendship, near-seated peers, classroom seating arrangements, primary school, proximity

## Abstract

The present study tests the hypothesis that friendships form on the basis of classroom seating proximity. Participants included 235 students (129 boys, 106 girls) in grades 3–5 (ages 8–11) who nominated friends at two time points (13–14 weeks apart). Teachers described seating arrangements. Concurrent analyses indicated that students sitting next to or nearby one another were more likely to receive friend nominations and be involved in reciprocated friendships than students seated elsewhere in the classroom. Longitudinal analyses indicated that classroom seating proximity was associated with the formation of new friendships. Most results for randomly selected outgoing friend nominations and randomly selected reciprocated friend dyads were replicated in analyses that included all friend nominations and all friend dyads.

## Introduction

Primary school children spend most of their days in assigned seats, in the company of classmates determined by a teacher. Do friendships form as a consequence of this proscribed proximity? College undergraduates report more “acquaintanceships” with those alphabetically assigned to adjacent seats than with those assigned seats farther away (Byrne and Buehler, 1955). Of course, outgoing acquaintance nominations should not be equated with friendships and there is no longitudinal evidence that seat proximity fosters friendship formation among children. The distinction matters because children spend far more time in classrooms than college students and have far less exposure to peers outside of class, so one would expect proximity to be particularly salient to friendship formation during primary school. The present investigation describes a naturalistic study of classroom seating in a primary school setting. We examine the degree to which proximity is associated with friend nominations and with participation in reciprocated friendships, concurrently and prospectively.

Friendships are critical to child wellbeing. Friends provide support and companionship, bolstering social skills and protecting against victimization (Bagwell and Bukowski, 2018). Friends shape the emotional lives of children, many of whom report feeling happiest when with friends (Hartup, 1996). Friends influence school achievement and behavioral adjustment (Laursen, 2018). Finally, friends are a developmental asset. Friendless children who make friends report declines in emotional problems; friended children who become friendless report increases in the same (Bukowski et al., 2010). Put simply, it is important that children have friends and it matters who those friends are.

The notion that physical proximity prompts friendship formation is not new. Researchers have long argued that most friendships arise from passive contact (Festinger et al., 1950). Mere exposure creates the impression of familiarity, which increases liking. Because attraction presumes similarity (Granberg and King, 1980), individuals gravitate to familiar others on the (accurate) assumption that homophily is a foundation for successful relationships (McPherson et al., 2001). Interpersonal contact theory holds that proximity also creates opportunities for rewarding interchanges that foster positive attitudes toward others (Allport, 1954). Experimental (Kahn and McGaughey, 1977) and quasi-experimental (Back et al., 2008) studies of college students indicate that attraction ratings are tied to seat proximity. Claims that classroom seat proximity fosters friendship formation, however, far outstrip research. In a study of primary school students, self-reports and peer reports of “hanging out” were associated with concurrent self-reports of sitting next to one another in class (Neal et al., 2014). One of the only longitudinal studies on the topic (Byrne, 1961) compared seat assignment rotations in three small (*n* < 35) college classes. Students assigned seat-neighbors higher (outgoing) friendship ratings than non-neighbors in the class that kept the same (alphabetically) assigned seats for 14 weeks, but not in classes where seat neighbors were constant for only 7 or 3.5 weeks.

Consider the implications for children, who have less freedom of movement and fewer options for friends than college students or even adolescents. In most primary school classrooms, teachers decide who sits next to whom and, by extension, who interacts with whom. We start from the premise that primary school children are classmates with most of their friends (Veenstra and Dijkstra, 2011). Given that interactions in classrooms are often constrained to near-seated peers, it follows that near-seated peers constitute the most likely pool of friend options. Some evidence ties classroom seating to likeability (a.k.a., social preference, peer ratings that range from *dislike very much* to *like very much*). In one study, closer seated classmates received higher concurrent likeability ratings than those seated farther away, but proximity did not predict prospective increases in likeability (van den Berg and Cillessen, 2015). In an experimental manipulation of seating assignments, moving children who disliked one another closer together raised the likeability scores of those who were most disliked, but (paradoxically) had no effect on their acceptance (liked-most) or rejection (liked-least) scores (van den Berg et al., 2012).

The present study examines the impact of proximity on friendship in a naturalistic, longitudinal study of primary school classrooms. We hypothesized that near-seated classmates were more likely to be friends than classmates seated farther away. We further predicted that after seat assignments changed, newly near-seated classmates were more likely to form friendships than classmates seated farther away.

## Methods

Participants included 235 children (129 boys, 106 girls) attending a public primary school required by statue to represent Florida students in terms of ethnicity and family income. The total included 59 3rd graders (*M* = 8.16 years, *SD* = 0.37), 87 4th graders (*M* = 9.17 years, *SD* = 0.44), and 89 5th graders (*M* = 10.20 years, *SD* = 0.48). Of this total, 42.6% were European-American, 27.7% were Hispanic-American, and 19.6% were African-American; the remainder identified other backgrounds.

### Procedure

Teacher consent, parent consent, and child assent were required for participation. Teachers received a gift card for participation. Data were collected in October 2019 and January 2020, an average of 13.7 (*SD* = 0.36) weeks apart. Teachers and students completed questionnaires the same day. Trained research assistants administered surveys to students on computer tablets in a quiet school setting. Teachers completed written surveys in the classroom, with no students present. The project was approved by “protocol #1355501.”

Of the 18 teachers invited to participate, 17 provided seat assignments at both time points. No teachers reported that friendships (keeping friends apart/together or promoting their formation) were a consideration, but 3 teachers indicated that students had some input into seat assignments. Students were assigned the same seat throughout the day (with the exception of lunch, recess, and P.E.). Nearly all teachers indicated that seat assignments at Time 1 were unchanged from the beginning of the school year, meaning that most students at Time 1 were seated in the same seats they were assigned when classes began. All classes reported seat assignment changes between Time 1 and Time 2. Students in most classes sat at tables (*N* = 14 classes); the remainder sat in rows (*N* = 3 classes). The average class size was 15 students (*SD* = 2.38, range = 11–19).

We invited 362 students from 17 classes to participate. Participation rates averaged 70.1% (*SD* = 8.0%; range = 60–82%). Given the small number of participants who did not name friends, the lack of systematic differences between those with and without friends, and the questionable logic of including students who failed to make friends in analyses of friendship formation, we excluded students without friends from concurrent analyses (*n* = 19) and longitudinal analyses (*n* = 25). The same pattern of results emerged when friendless students were included in analyses. Intraclass correlations (ρ), for binary variables when necessary, examined the proportion of variance in friendship, group and neighbor proximity, and Euclidean distance that could be attributed to the grouping structure (i.e., different classrooms). Low ICCs (within-classroom ρ = 0.00 to ρ = 0.05) indicated that observations within classrooms were no different than observations between classrooms (Hox, 1998; Heck et al., 2014), suggesting that classroom nestedness did not bias results.

### Measures

#### Friend Nominations

Students identified and rank-ordered an unlimited number of friends (“Who is your best friend/next best friend?”) from a roster of classmates. Same- and other-gender nominations were permitted. Students nominated an average of 4.73 (*SD* = 2.31) friends. Reciprocated friends nominated one another as friends. Students participated in an average of 3.04 (*SD* = 1.65) reciprocated friendships at Time 1 and 3.66 (*SD* = 1.53) at Time 2. Students made an average of 2.12 (*SD* = 1.34) new friend nominations at Time 2 and participated in an average of 1.15 (*SD* = 0.36) new reciprocated friendships at Time 2.

#### Proximity

**FIGURE 1:**
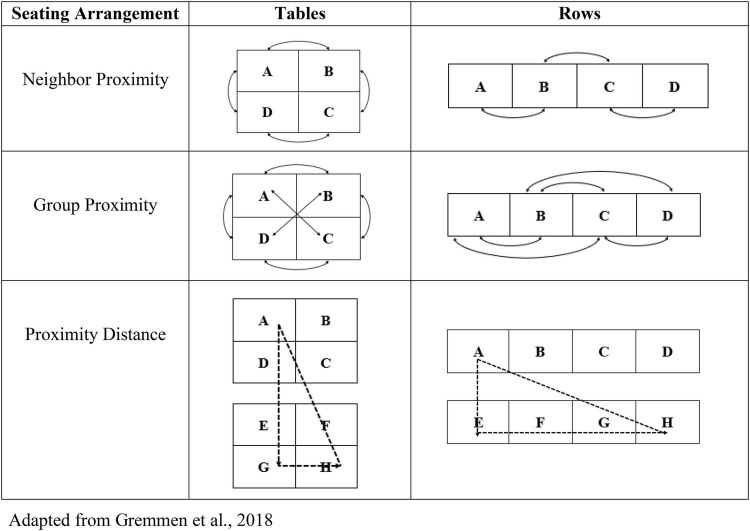
Illustration of three forms of propinquity [adapted from Gremmen et al. (2018)].

Teacher seating charts were used to calculate three forms of proximity for each pair of students in a classroom [see Figure 1, adapted from Gremmen et al. (2018)]. Neighbor proximity describes classmates seated directly beside one another in a row (i.e., A with B, B with C, and C with D) or at a table (i.e., A with B, and C with D), and those seated directly across from one another at a table (i.e., B with C, and D with A). Group proximity includes classmates identified as neighbors (see above) as well as those who were near neighbors; the latter were either one seat away in the same row (i.e., A with C, and B with D) or diagonal to one another at the same table (i.e., A with C, and B with D). Each pair of students received a dichotomous (yes or no) score for neighbor proximity and group proximity. Proximity distance describes the number of seats between two students, calculated as the squared Euclidean distance of the number of desks and rows between them (van den Berg et al., 2012). For example, if A and H are separated by 3 seats horizontally and 1 seat and 1 row vertically, the Euclidean distance is $\sqrt{\left(3^{2}+2^{2}\right)}=3.61$ Proximity distance scores were standardized within classrooms to account for differences in seating configurations.

We also calculated neighbor and group proximity change scores. Pairs of students were classified as “Getting Closer” if their seat assignments changed from not being neighbors or groupmates at Time 1 to becoming neighbors or groupmates at Time 2. Pairs of students were classified as “Staying Close” if they were neighbors or groupmates at Time 1 and Time 2. Pairs of students were classified as “Moving Apart” if their seat assignments changed from being neighbors or groupmates at Time 1 to not being neighbors or groupmates at Time 2. Pairs of students were classified as “Staying Apart” if they were not neighbors or groupmates at Time 1 and Time 2. The proximity distance change score represents the difference between the Time 2 distance score and the Time 1 distance score.

Note the assumptions underlying the different scores. Neighbor proximity assumes that sustained interactions required for friendship only arise with those in immediate proximity; other nearby peers (e.g., B with D) are assumed to be as disadvantaged as those who are seated at different tables or in different rows. Group proximity assumes that being seated at the same table or one seat away in a row is sufficiently close for sustained interactions required in a friendship; no distinctions are made between immediate neighbors and near-seated neighbors. Proximity distance assumes that every one unit increase in distance has an equivalent (linear) adverse impact on the interactions necessary for a friendship; no distinctions are made between those who are and are not seated at the same table or in the same row.

## Plan of Analysis

Four sets of logistic regressions were conducted in IBM SPSS (Version 26). Each included the same seven predictor variables in the first block: gender (same/other); ethnicity (same/other); grade in school; class size; teacher seating assignment strategy (student input/no student input); classroom layout (tables/rows); and the number of classmates a student nominated as a friend. The latter four variables were included to minimize differences between classrooms and individuals in opportunities for friendship formation. There were no two- or three-way interactions between any study variables, so interaction terms were omitted from the final analyses.

The first analyses examined concurrent associations between classroom proximity and outgoing friend nominations at Time 1. One of the three proximity measures was a predictor in the second block: neighbor proximity (yes/no); group proximity (yes/no); and proximity distance. The dependent variable was whether or not a student nominated a target as a friend. One outgoing friend nomination from each student (*N* = 235) was randomly selected for the analyses, with the stipulation that each student be included once (and only once) as a nominator and once (and only once) as a target. In 4 cases, the same two children occupied the roles of both target and nominator (A was nominator with B as target and B was nominator with A as target). The same pattern of results emerged when these children were assigned different targets. Randomly selected pairings are our primary focus, because randomization avoids problems arising from the use of the entire dataset in the analyses, particularly bias arising from an inflated N and unequal contributions from participants who made a widely varying number of friend nominations. To replicate the results and address concerns that the random sample may not be representative of the larger group, we conducted identical supplemental analyses on all outgoing Time 1 friend nominations (*N* = 3,375). In these supplemental analyses, each student could appear multiple times as a nominator (*M* = 13.21, *SD* = 3.48, range = 8–18) and as a target (*M* = 13.29, *SD* = 2.78, range = 8–18).

The second analyses examined concurrent associations between proximity and participation in reciprocated friendships at Time 1. One of the three proximity measures was a predictor in the second block: neighbor proximity (yes/no); group proximity (yes/no); and proximity distance. The dependent variable was whether or not both students in a dyad reciprocally nominated one another friends. Each student was included in one (and only one) randomly selected dyad (*N* = 114 dyads). Identical supplemental analyses included all dyads (*N* = 1,165). In these supplemental analyses, each student could appear in multiple dyads (*M* = 9.91, *SD* = 2.84, range = 3–17). Unilateral friends (outgoing nominations that were not reciprocated) were omitted; identical results emerged from analyses with unilateral friends classified as non-friends.

The third analyses examined longitudinal associations between changes in proximity from Time 1 to Time 2 and new outgoing friend nominations at Time 2. One of the three proximity change measures was a predictor in the second block: Neighbor proximity (staying close and getting closer vs. staying apart and moving apart); group proximity (staying close and getting closer vs. staying apart and moving apart); and proximity distance. The dependent variable was whether or not a student nominated a target as a new friend at Time 2. One outgoing friend nomination from each student (*N* = 228) was randomly selected for the analyses, with the stipulation that (a) each student be included once (and only once) as nominator and once (and only once) as target and (b) targets were not nominated as friends at Time 1. In one case, the target identified the nominator as a unilateral friend at Time 1; the same pattern of results emerged when this student was omitted from the analyses. In 6 cases, the same two children occupied the roles of both target and nominator (A was nominator with B as target and B was nominator with A as target). The same pattern of results emerged when these children were assigned different targets. Identical supplemental analyses included all outgoing Time 2 friend nominations (*N* = 1,938). In these supplemental analyses, each student could appear multiple times as a nominator (*M* = 8.48, *SD* = 3.01, range = 2–17) and as a target (*M* = 8.48, *SD* = 3.20, range = 1–17). In 384 cases, a target identified the nominator as a unilateral friend at Time 1; the same pattern of results emerged when these students were omitted from supplemental analyses.

The fourth analyses examined longitudinal associations between changes in proximity from Time 1 to Time 2 and the establishment of new reciprocated friendships at Time 2. One of the three proximity change measures was a predictor in the second block: Neighbor proximity (staying close and getting closer vs. staying apart and moving apart); group proximity (staying close and getting closer vs. staying apart and moving apart); and proximity distance. The dependent variable was whether or not both students in the dyad reciprocally nominated one another as friends at Time 2. Each student was included in one (and only one) randomly selected dyad, with the stipulation that neither student nominated the other as a friend at Time 1 (*N* = 91 dyads). Identical supplemental analyses included all Time 1 non-friend dyads (*N* = 586 dyads). In these supplemental analyses, each student could appear in multiple dyads (*M* = 5.13, *SD* = 2.93, range = 1–14). Unilateral friends were omitted; identical results emerged from analyses with unilateral friends classified as non-friends.

There were no statistically significant differences between those who did and did not make friend nominations and between those who were and were not included in analyses on any demographic or proximity variable. Little’s MCAR test indicated that data were missing completely at random, χ^2^(2) = 3.04, *p* = 0.22.

## Results

### Concurrent Associations Between Seat Assignment Proximity and Friendships

#### Outgoing Friend Nominations

Table 1 presents results from analyses that examined concurrent associations between seat proximity and outgoing friend nominations at Time 1. Gender, grade, class size, and number of nominations made were associated with outgoing friend nominations. Randomly selected same-gender targets were more likely than other-gender targets to be nominated as friends. As grade and class size decreased and the number of friend nominations increased, randomly selected targets were more likely to be nominated as friends. Neighbor proximity, group proximity, and proximity distance were associated with outgoing friend nominations. Odds-ratios indicate that randomly selected targets seated nearby were 3–5 times more likely to be nominated as friends than those who were not seated nearby.

**TABLE 1:** Concurrent logistic regressions predicting Time 1 outgoing friend nominations from Time 1 classroom seating: One randomly selected nomination per student.

	Neighbor proximity				Group proximity				Proximity distance			
Variable	β	(SE)	*p*	OR (95% CI)	β	(SE)	*p*	OR (95% CI)	β	(SE)	*p*	OR (95% CI)
**Block 1**												
Dyad gender	−1.75	(0.34)	0.001	0.17 (0.09, 0.34)	−1.75	(0.34)	0.001	0.17 (0.09, 0.34)	−1.75	(0.34)	0.001	0.17 (0.09, 0.34)
Dyad ethnicity	0.22	(0.35)	0.530	1.24 (0.63, 2.46)	0.22	(0.35)	0.530	1.24 (0.63, 2.46)	0.22	(0.35)	0.530	1.24 (0.63, 2.46)
Grade	−0.49	(0.25)	0.045	0.61 (0.38, 0.99)	−0.49	(0.25)	0.045	0.61 (0.38, 0.99)	−0.49	(0.25)	0.045	0.61 (0.38, 0.99)
Class size	−0.22	(0.09)	0.012	0.80 (0.68, 0.95)	−0.22	(0.09)	0.012	0.80 (0.68, 0.95)	−0.22	(0.09)	0.012	0.80 (0.68, 0.95)
Teacher seating strategy	−0.85	(0.48)	0.075	0.43 (0.17, 1.09)	−0.85	(0.48)	0.075	0.43 (0.17, 1.09)	−0.85	(0.48)	0.075	0.43 (0.17, 1.09)
Classroom layout	0.68	(0.46)	0.141	1.97 (0.80, 4.86)	0.68	(0.46)	0.141	1.97 (0.80, 4.86)	0.68	(0.46)	0.141	1.97 (0.80, 4.86)
Friends nominated	0.58	(0.18)	0.001	1.78 (1.25, 2.54)	0.58	(0.18)	0.001	1.78 (1.25, 2.54)	0.58	(0.18)	0.001	1.78 (1.25, 2.54)
*r*^2^ for step	0.32				0.32				0.32			
**Block 2**												
Neighbor proximity	1.61	(0.55)	0.003	5.00 (1.71, 14.65)								
Group proximity					1.11	(0.45)	0.014	3.03 (1.25, 7.34)				
Proximity distance									−0.41	(0.16)	0.011	0.66 (0.49, 0.91)
Total *r*^2^	0.36				0.34				0.35			
χ^2^ (df) *p*	70.50 (8)		0.001		67.43 (8)		0.001		68.17 (8)		0.001	

*N = 235. Unstandardized beta weights and 95% confidence intervals for odds ratios are reported. Nominator gender: 1 = same gender, 2 = other gender. Dyad ethnicity: 1 = same ethnicity, 2 = other ethnicity. Teacher seating strategy: 1 = no student input, 2 = student input. Classroom layout: 1 = tables, 2 = rows.*

Supplementary Table 1 presents results from analyses that included all outgoing friend nominations. Consistent with findings for randomly selected friend nominations, neighbor proximity, group proximity, and proximity distance were associated with outgoing friend nominations.

#### Reciprocated Friendships

Table 2 presents results from analyses that examined concurrent associations between seat proximity and participation in reciprocated friendships at Time 1. Gender, neighbor proximity, group proximity, and proximity distance were associated with reciprocated friendships. Randomly selected same-gender dyads were more likely than other-gender dyads to be reciprocated friends. Randomly selected dyads seated near one another were 12–18 times more likely to be reciprocated friends than those seated farther away.

**TABLE 2:** Concurrent logistic regressions predicting Time 1 reciprocated friendships from Time 1 classroom seating: One randomly selected dyad per student.

	Neighbor proximity				Group proximity				Proximity distance			
Variable	β	(SE)	*p*	OR (95% CI)	β	(SE)	*p*	OR (95% CI)	β	(SE)	*p*	OR (95% CI)
**Block 1**												
Dyad gender	−2.63	(0.57)	0.001	0.07 (0.23, 0.22)	−2.63	(0.57)	0.001	0.07 (0.23, 0.22)	−2.63	(0.57)	0.001	0.07 (0.23, 0.22)
Dyad ethnicity	−0.95	(0.61)	0.116	0.38 (0.12, 1.27)	−0.95	(0.61)	0.116	0.38 (0.12, 1.27)	−0.95	(0.61)	0.116	0.38 (0.12, 1.27)
Grade	0.19	(0.38)	0.614	1.21 (0.58, 2.55)	0.19	(0.38)	0.614	1.21 (0.58, 2.55)	0.19	(0.38)	0.614	1.21 (0.58, 2.55)
Class size	−0.16	(0.15)	0.268	0.85 (0.64, 1.13)	−0.16	(0.15)	0.268	0.85 (0.64, 1.13)	−0.16	(0.15)	0.268	0.85 (0.64, 1.13)
Teacher seating strategy	−0.49	(0.74)	0.510	0.61 (0.14, 2.62)	−0.49	(0.74)	0.510	0.61 (0.14, 2.62)	−0.49	(0.74)	0.510	0.61 (0.14, 2.62)
Classroom layout	0.32	(0.74)	0.668	1.37 (0.32, 5.85)	0.32	(0.74)	0.668	1.37 (0.32, 5.85)	0.32	(0.74)	0.668	1.37 (0.32, 5.85)
Friends nominated	0.92	(0.31)	0.002	2.52 (1.38, 4.57)	0.92	(0.31)	0.002	2.52 (1.38, 4.57)	0.92	(0.31)	0.002	2.52 (1.38, 4.57)
*r*^2^ for step	0.48				0.48				0.48			
**Block 2**												
Neighbor proximity	2.88	(1.1)	0.009	17.80 (2.06,153.8)								
Group proximity					2.52	(0.82)	0.002	12.49 (2.52, 61.8)				
Proximity distance									−0.72	(0.31)	0.021	0.48 (0.26, 0.90)
Total *r*^2^	0.55				0.57				0.53			
χ^2^ (df) *p*	57.76 (8)		0.001		60.20 (8)		0.001		54.60 (8)		0.001	

*N = 114 dyads. Unstandardized beta weights and 95% confidence intervals for odds ratios are reported. Dyad gender: 1 = same gender, 2 = other gender. Dyad ethnicity: 1 = Same ethnicity, 2 = different ethnicity. Teacher seating strategy: 1 = no student input, 2 = student input. Classroom layout: 1 = tables, 2 = rows.*

Supplementary Table 2 presents results for analyses that involved all friendship dyads. Consistent with findings from the randomly selected subsample, neighbor proximity (borderline) and group proximity were associated with reciprocated friendships; Unlike findings from the randomly selected subsample, proximity distance was unrelated to reciprocated friendships.

### Longitudinal Associations Between Seat Assignment Proximity and New Friendships

#### Outgoing Friend Nominations

Table 3 presents results from analyses that examined longitudinal associations between changes in seat proximity from Time 1 to Time 2 and new outgoing friend nominations at Time 2. Of those not nominated as friends at Time 1, randomly selected same-gender targets were more likely than other-gender targets to be nominated as friends. Changes in neighbor proximity, group proximity, and proximity distance were associated with new outgoing friend nominations. Of those not nominated as friends at Time 1, randomly selected targets whose seats became or remained close were about 8–10 times more likely to be nominated as friends at Time 2 than those whose seats became or remained far apart.

**TABLE 3:** Longitudinal logistic regressions predicting new Time 2 outgoing friend nominations from changes in classroom seating: One randomly selected nomination per student.

	Neighbor proximity				Group proximity				Proximity distance			
Variable	β	(SE)	*p*	OR (95% CI)	β	(SE)	*p*	OR (95% CI)	β	(SE)	*p*	OR (95% CI)
**Block 1**												
Dyad gender	−0.95	(0.30)	0.002	0.38 (0.22, 0.70)	−0.95	(0.30)	0.002	0.38 (0.22, 0.70)	-0.95	(0.30)	0.002	0.38 (0.22, 0.70)
Dyad ethnicity	0.40	(0.34)	0.906	1.04 (0.54, 2.01)	0.40	(0.34)	0.906	1.04 (0.54, 2.01)	0.40	(0.34)	0.906	1.04 (0.54, 2.01)
Grade	−0.18	(0.22)	0.422	0.84 (0.54, 1.29)	−0.18	(0.22)	0.422	0.84 (0.54, 1.29)	−0.18	(0.22)	0.422	0.84 (0.54, 1.29)
Class size	−0.13	(0.08)	0.112	0.88 (0.76, 1.03)	−0.13	(0.08)	0.112	0.88 (0.76, 1.03)	−0.13	(0.08)	0.112	0.88 (0.76, 1.03)
Teacher seating strategy	0.11	(0.40)	0.794	1.11 (0.50, 2.45)	0.11	(0.40)	0.794	1.11 (0.50, 2.45)	0.11	(0.40)	0.794	1.11 (0.50, 2.45)
Classroom layout	0.37	(0.42)	0.382	1.44 (0.63, 3.29)	0.37	(0.42)	0.382	1.44 (0.63, 3.29)	0.37	(0.42)	0.382	1.44 (0.63, 3.29)
Friends nominated	−0.45	(0.15)	0.003	0.64 (0.47, 0.86)	−0.45	(0.15)	0.003	0.64 (0.47, 0.86)	−0.45	(0.15)	0.003	0.64 (0.47, 0.86)
*r*^2^ for step	0.16				0.16				0.16			
**Block 2**												
Neighbor proximity	2.34	(0.53)	0.001	10.41 (3.72, 29.14)								
Group proximity					2.13	(0.46)	0.001	8.43 (3.45, 20.66)				
Proximity distance change									−0.47	(0.12)	0.001	0.62 (0.49, 0.79)
Total *r*^2^	0.28				0.29				0.25			
χ^2^ (df) *p*	52.04 (8)	0.001		53.94 (8)	0.001			46.20 (8)		0.001		

*N = 228. Unstandardized beta weights and 95% confidence intervals for odds ratios are reported. Dyad gender: 1 = same gender, 2 = other gender. Dyad ethnicity: 1 = same ethnicity, 2 = other ethnicity. Teacher seating strategy: 1 = no child input, 2 = child input. Classroom layout: 1 = tables, 2 = rows. Neighbor proximity and group proximity: 1 = moving apart (n = 11 new non-neighbors; n = 15 new non-groupmates) or staying apart (n = 190 stable non-neighbors; n = 179 stable non-groupmates), 2 = getting closer (n = 23 new neighbors; n = 29 new groupmates) or staying close (n = 4 stable neighbors; n = 5 stable groupmates).*

Similar results emerged from analyses that included all outgoing friend nominations (Supplementary Table 3). Neighbor proximity, group proximity, and proximity distance were associated with new outgoing friend nominations.

Additional analyses excluding the Staying Close group (*n* = 4–5 for randomly selected nominations and *n* = 24–35 for all nominations) revealed the same pattern of statistically significant results in comparisons of the Getting Closer group with the Staying Apart/Moving Apart group.

#### Reciprocated Friendships

Table 4 presents results from analyses that examined longitudinal associations between changes in seat proximity from Time 1 to Time 2 and participation in new reciprocated friendships at Time 2. Changes in neighbor proximity, group proximity, and proximity distance were associated with new reciprocated friendships. Of those who were not reciprocated friends at Time 1, randomly selected dyads whose seats became or remained close were 32–46 times more likely to be reciprocated friends at Time 2 than those whose seats became or remained far apart.

**TABLE 4:** Longitudinal logistic regressions predicting new Time 2 reciprocated friendships from changes in classroom seating: One randomly selected dyad per student.

	Neighbor proximity				Group proximity				Proximity distance			
Variable	β	(SE)	*P*	OR (95% CI)	β	(SE)	*p*	OR (95% CI)	β	(SE)	*p*	OR (95% CI)
**Block 1**												
Dyad gender	−1.38	(0.74)	0.061	0.25 (0.06, 1.06)	−1.38	(0.74)	0.061	0.25 (0.06, 1.06)	−1.38	(0.74)	0.061	0.25 (0.06, 1.06)
Dyad ethnicity	0.56	(0.81)	0.485	1.76 (0.36, 8.55)	0.56	(0.81)	0.485	1.76 (0.36, 8.55)	0.56	(0.81)	0.485	1.76 (0.36, 8.55)
Grade	−0.56	(0.57)	0.325	0.57 (0.19, 1.75)	−0.56	(0.57)	0.325	0.57 (0.19, 1.75)	−0.56	(0.57)	0.325	0.57 (0.19, 1.75)
Class size	0.02	(0.19)	0.925	1.02 (0.69, 1.49)	0.02	(0.19)	0.925	1.02 (0.69, 1.49)	0.02	(0.19)	0.925	1.02 (0.69, 1.49)
Teacher seating strategy	−0.05	(0.93)	0.961	0.95 (0.15, 5.92)	−0.05	(0.93)	0.961	0.95 (0.15, 5.92)	−0.05	(0.93)	0.961	0.95 (0.15, 5.92)
Classroom layout	0.55	(1.02)	0.587	1.74 (0.24, 12.76)	0.55	(1.02)	0.587	1.74 (0.24, 12.76)	0.55	(1.02)	0.587	1.74 (0.24, 12.76)
Friends nominated	−0.07	(0.34)	0.826	0.93 (0.47, 1.82)	−0.07	(0.34)	0.826	0.93 (0.47, 1.82)	−0.07	(0.34)	0.826	0.93 (0.47, 1.82)
*r*^2^ for step	0.10				0.10				0.10			
**Block 2**												
Neighbor proximity	3.49	(1.02)	0.001	32.91 (4.42, 244.81)								
Group proximity					3.84	(1.14)	0.001	46.36 (4.91, 437.67)				
Proximity distance change									−1.35	(0.40)	0.001	0.26 (0.12, 0.56)
Total *r*^2^	0.40			0.44			0.44					
χ^2^ (df) *p*	20.41 (8)	0.001		22.38 (8)	0.004		22.69 (8)	0.001				

*N = 91 dyads. Unstandardized beta weights and 95% confidence intervals for odds ratios are reported. Dyad gender: 1 = same gender, 2 = other gender. Dyad ethnicity: 1 = Same ethnicity, 2 = different ethnicity. Teacher seating strategy: 1 = no student input, 2 = student input. Classroom layout: 1 = tables, 2 = rows. Neighbor proximity and group proximity: 1 = moving apart (n = 12 new non-neighbors; n = 12 new non-groupmates) or staying apart (n = 64 stable non-neighbors; n = 62 stable non-groupmates), 2 = getting closer (n = 11 new neighbors; n = 11 new groupmates) or staying close (n = 4 stable neighbors; n = 6 stable groupmates).*

Similar results emerged from analyses that included all dyads in the (Supplementary Table 4). Neighbor proximity, group proximity, and proximity distance (borderline) were associated with new reciprocated friendships.

Additional analyses excluding the Staying Close group (*n* = 4–6 for randomly selected dyads and *n* = 8–11 for all dyads) revealed the same pattern of statistically significant results in comparisons of the Getting Closer group with the Staying Apart/Moving Apart group.

## Discussion

Friendships reflect classroom seat assignments. Students sitting next to or nearby one another were more likely to be friends than students seated elsewhere in the classroom. Moreover, seat assignment changes were associated with the formation of new friendships. Students were more likely to become friends with newly near-seated classmates than with those who remained or became seated farther away.

The findings suggest that friendships with classmates are not exclusively predicated on exposure. Recall that repeated exposure creates the impression of familiarity, which Festinger et al. (1950) hypothesized as the foundation for attraction. Primary school students in the same class do not lack for exposure to one another: The students in this study spent most of every day with the same 15 or so classmates. By the middle of the school year, there were no unfamiliar peers. Yet when seat assignments changed, new seatmates were apt to become new friends, consistent with claims that mere exposure may be a necessary condition for friendship but it is not sufficient. Instead, proximity transcends familiarity by providing opportunities for the kind of exchanges that undergird friendships (Allport, 1954). One possibility is that proximity is necessary to identify rewarding interaction partners (Byrne, 1961). Proximity may also narrow the field of friend options, creating a small pool from which children choose the most compatible mate. Alternatively, children may pragmatically befriend proximal affiliates. School is more fun and classwork is more successful in the company of a friend (Hartl et al., 2015). In this scenario, common ground activities (e.g., dyadic seatwork) can serve as the basis for friendship formation (Gottman and Graziano, 1983).

Most teachers focus on academic considerations when assigning seats (Gremmen et al., 2016). With good reason: Near-seated classmates influence student achievement and engagement (Gremmen et al., 2018). Although less common, seat assignments based on social motives have been linked to concurrent peer status (Gest and Rodkin, 2011). The present study is the first to demonstrate that primary school seat assignments foster new friendships. Our findings are less equivocal than those from college classrooms (Byrne, 1961), which makes sense given that mobility constrains the social options of children but not young adults. Of course, students were not glued to their seats; interactions with far-seated peers undoubtedly occurred during lunch, recess and (in some classes) free time activities. The fact that new friends tended to emerge among the newly near-seated—despite opportunities for engagement with other classmates—underscores the power of proximity in friendship formation. Taken together, the findings highlight the enormous influence that teachers wield over the interpersonal lives of children. With great power comes great responsibility. We urge teachers to exercise their power judiciously, because iatrogenic effects were observed in randomized control trials designed to reduce antipathies (Braun et al., 2020) and increase peer acceptance of aggressive students (van den Berg and Stoltz, 2018).

Results from the full sample supplemental analyses did not perfectly replicate those from the randomly selected samples. Findings for group proximity were the most robust, suggesting that children are willing (and able) to overlook their nearest neighbors in favor of those seated close enough for sustained communication. Discrepancies between the random sample and the full sample involved concurrent, not longitudinal data. *Post hoc* explanations should be viewed with caution, but from a statistical standpoint the effect sizes from the concurrent analyses were smaller than those from the longitudinal analyses, probably because concurrent friendships include affiliations that began prior to the start of the school year, withstanding the vagaries of seat placement. Instances where supplemental analyses failed to replicate may be traced to the inability to control for non-independence in the total sample, thereby missing the relatively small effects identified in the random sample.

Our study is not without limitations. Undoubtedly, some students were friends the previous year and some of these friends were seated nearby, a potential confound for the concurrent but not the longitudinal analyses. Seat assignments were not random. Most teachers indicated that students had no input in seat selection and all teachers indicated that friendship was not a factor, but we cannot rule out the possibility that interpersonal concerns implicitly informed decisions about seating. Teachers did not assign seats with an eye toward fostering friendships, but we suspect that many avoided placing children who disliked one another in close proximity. Assuming that antipathies are based on dissimilarities (Laursen et al., 2010), this practice would increase the chances that near-seated peers share commonalities. Nor can we rule out the possibility that teachers assigned seats by ability, which formed the basis of new friendships. The findings do not discount the role of similarity in friendship formation (Laursen, 2017). Among those who became near seated, children undoubtedly exercised a preference for the most compatible options. Some will object that multilevel models were not employed. We concede that our design cannot detect classroom differences, but argue that this is hardly a fatal flaw; we suspect that differences (should they arise) reflect teacher classroom management practices, which have yet to be considered in studies of seat assignment effects. Further, our analyses included control variables that (imperfectly) examined class distinctions and addressed concerns about the fact that some students made more friend nominations–and participated in more friend dyads–than others. Many more classes with many more students are required for multilevel modeling (McNeish and Stapleton, 2016). Dyad-level non-independence is a consideration, but round-robin analyses (i.e., SOREMO) cannot be conducted with dichotomous outcomes (Kenny, 1998). To mitigate non-independence concerns, the primary analyses limited students to participation in a single dyad (in the case of randomly selected reciprocated friends) or to participation once as a nominator and once as a target (in the case of randomly selected friend nominations). The study draws strength from this use of random assignment, which can help minimize confounds and eliminate alternative explanations, even with quasi-experimental data (Gonzalez et al., 2012). Due to the vagaries of random assignment, a small number of students were over-represented (i.e., nested within the same relationship) but the same results emerged when they were reassigned to other partners.

Classroom proximity assumes outsized importance during the primary school years because children this age have few other sustained opportunities to meet (and engage with) friends and because companionship is central to the definition of friendship (Buhrmester and Furman, 1987). We have long known that most children report that most of their friends are in the same classroom (Smith and Inder, 1990). We now know that they are probably seated nearby.

## Data Availability Statement

The raw data supporting the conclusions of this article will be made available by the authors, without undue reservation.

## Ethics Statement

The studies involving human participants were reviewed and approved by the Institutional Review Board of Florida Atlantic University (protocol #1355501). Written informed consent to participate in this study was provided by the participants’ legal guardian/next of kin.

## Author Contributions

BL and SF contributed to the conception and design of the study. SF organized the data set, performed the statistical analyses, and wrote the original draft. BL supervised the project, reviewed, and edited. Both authors contributed to the final manuscript.

## Conflict of Interest

The authors declare that the research was conducted in the absence of any commercial or financial relationships that could be construed as a potential conflict of interest.

## Publisher’s Note

All claims expressed in this article are solely those of the authors and do not necessarily represent those of their affiliated organizations, or those of the publisher, the editors and the reviewers. Any product that may be evaluated in this article, or claim that may be made by its manufacturer, is not guaranteed or endorsed by the publisher.
