# Supplementary material for: Classroom Seat Proximity Predicts Friendship Formation
Source: Front Psychol. 2022 May 3;13:796002. doi: 10.3389/fpsyg.2022.796002 (PMC9113197; doi:10.3389/fpsyg.2022.796002)
Supplement: Supplementary file 1 [file Data_Sheet_1.pdf]

Supplemental Table S1

Concurrent logistic regressions predicting Time 1 outgoing friend nominations from Time 1 classroom seating: All nominations (Entire Sample)

	Neighbor Proximity				Group Proximity				Proximity Distance			
Variable	β	(SE)	*p*	OR (95% CI)	β	(SE)	*p*	OR (95% CI)	β	(SE)	*p*	OR (95% CI)
Block 1												
Dyad Gender	-1.98	(.09)	.001	0.14 (0.12, 0.16)	-1.98	(.09)	.001	0.14 (0.12, 0.16)	-1.98	(.09)	.001	0.14 (0.12, 0.16)
Dyad Ethnicity	-0.13	(.09)	.143	0.87 (0.73, 1.05)	-0.13	(.09)	.143	0.87 (0.73, 1.05)	-0.13	(.09)	.143	0.87 (0.73, 1.05)
Grade	-0.06	(.06)	.329	0.94 (0.83, 1.06)	-0.06	(.06)	.329	0.94 (0.83, 1.06)	-0.06	(.06)	.329	0.94 (0.83, 1.06)
Class Size	-0.05	(.02)	.017	0.95 (0.91, 0.99)	-0.05	(.02)	.017	0.95 (0.91, 0.99)	-0.05	(.02)	.017	0.95 (0.91, 0.99)
Teacher Seating Strategy	-0.38	(.12)	.001	0.68 (0.54, 0.86)	-0.38	(.12)	.001	0.68 (0.54, 0.86)	-0.38	(.12)	.001	0.68 (0.54, 0.86)
Classroom Layout	0.01	(.11)	.930	1.01 (0.81, 1.26)	0.01	(.11)	.930	1.01 (0.81, 1.26)	0.01	(.11)	.930	1.01 (0.81, 1.26)
Friends Nominated	0.79	(.05)	.001	2.20 (1.99, 2.43)	0.79	(.05)	.001	2.20 (1.99, 2.43)	0.79	(.05)	.001	2.20 (1.99, 2.43)
r^2 for step	.31				.31				.31			
Block 2												
Neighbor Proximity	0.44	(.15)	.003	1.56 (1.16, 2.01)								
Group Proximity					0.56	(.13)	.001	1.75 (1.36, 2.26)				
Proximity Distance									-0.14	(.04)	.001	0.87 (0.80, 0.95)
Total r^2	.32				.32				.32			
χ^2 (df) *p*	883.22 (8)		.001		893.40 (8)		.001		885.31 (8)		.001	

Notes. *N*=3375 (entire sample). Unstandardized beta weights and 95% confidence intervals for odds ratios are reported. Dyad gender: 1=same gender, 2=other gender. Dyad ethnicity: 1=same ethnicity, 2=other ethnicity. Teacher seating strategy: 1=no student input, 2=student input. Classroom layout: 1=tables, 2=rows.

Supplemental Table S2

Concurrent logistic regressions predicting Time 1 reciprocated friendships from Time 1 classroom seating: All dyads (Entire Sample)

	Neighbor Proximity				Group Proximity				Proximity Distance			
Variable	β	(SE)	*p*	OR (95% CI)	β	(SE)	*p*	OR (95% CI)	β	(SE)	*p*	OR (95% CI)
Block 1												
Dyad Gender	-3.01	(.19)	.001	0.05 (0.03, 0.07)	-3.01	(.19)	.001	0.05 (0.03, 0.07)	-3.01	(.19)	.001	0.05 (0.03, 0.07)
Dyad Ethnicity	0.06	(.18)	.716	1.07 (0.75, 1.50)	0.06	(.18)	.716	1.07 (0.75, 1.50)	0.06	(.18)	.716	1.07 (0.75, 1.50)
Grade	0.30	(.12)	.796	1.03 (0.81, 1.32)	0.30	(.12)	.796	1.03 (0.81, 1.32)	0.30	(.12)	.796	1.03 (0.81, 1.32)
Class Size	-0.30	(.05)	.001	0.77 (0.69, 0.84)	-0.30	(.05)	.001	0.77 (0.69, 0.84)	-0.30	(.05)	.001	0.77 (0.69, 0.84)
Teacher Seating Strategy	-0.08	(.25)	.757	0.93 (0.57, 1.51)	-0.08	(.25)	.757	0.93 (0.57, 1.51)	-0.08	(.25)	.757	0.93 (0.57, 1.51)
Classroom Layout	0.06	(.23)	.784	1.06 (0.68, 1.67)	0.06	(.23)	.784	1.06 (0.68, 1.67)	0.06	(.23)	.784	1.06 (0.68, 1.67)
Friends Nominated	0.39	(.04)	.001	1.48 (1.38, 1.60)	0.39	(.04)	.001	1.48 (1.38, 1.60)	0.39	(.04)	.001	1.48 (1.38, 1.60)
r^2 for step	.50				.50				.50			
Block 2												
Neighbor Proximity	0.54	(.31)	.080	1.72 (0.94, 3.16)								
Group Proximity					0.69	(.27)	.010	1.99 (1.18, 3.35)				
Proximity Distance									-0.13	(.08)	.120	0.88 (0.74, 1.03)
Total r^2	.51				.51				.51			
χ^2 (df) *p*	516.88 (8)		.001		520.48 (8)		.001		516.29 (8)		<.001	

Notes. *N*=1165 dyads (entire sample). Unstandardized beta weights and 95% confidence intervals for odds ratios are reported. Dyad gender: 1=same gender, 2=other gender. Dyad ethnicity: 1=Same ethnicity, 2=different ethnicity. Teacher seating strategy: 1=no student input, 2=student input. Classroom layout: 1=tables, 2=rows.

Supplemental Table S3

Longitudinal logistic regressions predicting new Time 2 outgoing friend nominations from changes in classroom seating: All nominations (Entire Sample)

	Neighbor Proximity				Group Proximity				Proximity Distance			
Variable	β	(SE)	*p*	OR (95% CI)	β	(SE)	*p*	OR (95% CI)	β	(SE)	*p*	OR (95% CI)
Block 1												
Dyad Gender	-1.18	(.12)	.001	0.31 (0.24, 0.39)	-1.18	(.12)	.001	0.31 (0.24, 0.39)	-1.18	(.12)	.001	0.31 (0.24, 0.39)
Dyad Ethnicity	-0.09	(.13)	.473	0.92 (0.72, 1.17)	-0.09	(.13)	.473	0.92 (0.72, 1.17)	-0.09	(.13)	.473	0.92 (0.72, 1.17)
Grade	0.06	(.09)	.501	1.06 (0.89, 1.27)	0.06	(.09)	.501	1.06 (0.89, 1.27)	0.06	(.09)	.501	1.06 (0.89, 1.27)
Class Size	-0.19	(.03)	.001	0.83 (0.78, 0.88)	-0.19	(.03)	.001	0.83 (0.78, 0.88)	-0.19	(.03)	.001	0.83 (0.78, 0.88)
Teacher Seating Strategy	-0.07	(.16)	.645	0.93 (0.68, 1.27)	-0.07	(.16)	.645	0.93 (0.68, 1.27)	-0.07	(.16)	.645	0.93 (0.68, 1.27)
Classroom Layout	0.19	(.17)	.256	1.21 (0.87, 1.67)	0.19	(.17)	.256	1.21 (0.87, 1.67)	0.19	(.17)	.256	1.21 (0.87, 1.67)
Friends Nominated	-0.18	(.06)	.002	0.84 (0.75, 0.94)	-0.18	(.06)	.002	0.84 (0.75, 0.94)	-0.18	(.06)	.002	0.84 (0.75, 0.94)
r^2 for step	.13				.13				.13			
Block 2												
Neighbor Proximity	0.78	(.19)	.001	0.83 (0.74, 0.93)								
Group Proximity					0.65	(.17)	.001	1.92 (1.39, 2.66)				
Proximity Distance Change									-0.12	(.05)	.008	0.89 (0.81, 0.97)
Total r^2	.14				.14				.13			
χ^2 (df) *p*	175.75 (8)		.001		175.49 (8)		.001		167.91 (8)		.001	

Notes. *N*=1938 (entire sample). Unstandardized beta weights and 95% confidence intervals for odds ratios are reported are reported. Dyad gender: 1=same gender, 2=other gender. Dyad ethnicity: 1=same ethnicity, 2=other ethnicity. Teacher seating strategy: 1=no child input, 2=child input. Classroom Layout: 1=tables, 2=rows. Neighbor proximity and group proximity: 1=moving apart (*n*=134 new non-neighbors; *n*=171 new non-groupmates) or staying apart (*n*=1640 stable non-neighbors; *n*=1530 stable non-groupmates), 2=getting closer (*n*=140 new neighbors; *n*=202 new groupmates) or staying close (*n*=24 stable neighbors; *n*=35 stable groupmates).

Supplemental Table S4

Longitudinal logistic regressions predicting new Time 2 reciprocated friendships from changes in classroom seating: All dyads (Entire Sample)

	Neighbor Proximity				Group Proximity				Proximity Distance			
Variable	β	(SE)	*p*	OR (95% CI)	β	(SE)	*p*	OR (95% CI)	β	(SE)	*p*	OR (95% CI)
Block 1												
Dyad Gender	-2.20	(.38)	.001	0.11 (0.05, 0.23)	-2.20	(.38)	.001	0.11 (0.05, 0.23)	-2.20	(.38)	.001	0.11 (0.05, 0.23)
Dyad Ethnicity	-0.18	(.40)	.645	0.83 (0.38, 1.82)	-0.18	(.40)	.645	0.83 (0.38, 1.82)	-0.18	(.40)	.645	0.83 (0.38, 1.82)
Grade	-0.04	(.27)	.876	0.96 (0.56, 1.63)	-0.04	(.27)	.876	0.96 (0.56, 1.63)	-0.04	(.27)	.876	0.96 (0.56, 1.63)
Class Size	-0.17	(.10)	.087	0.85 (0.70, 1.02)	-0.17	(.10)	.087	0.85 (0.70, 1.02)	-0.17	(.10)	.087	0.85 (0.70, 1.02)
Teacher Seating Strategy	-0.11	(.46)	.807	0.89 (0.36, 2.20)	-0.11	(.46)	.807	0.89 (0.36, 2.20)	-0.11	(.46)	.807	0.89 (0.36, 2.20)
Classroom Layout	-0.10	(.52)	.843	0.90 (0.32, 2.51)	-0.10	(.52)	.843	0.90 (0.32, 2.51)	-0.10	(.52)	.843	0.90 (0.32, 2.51)
Friends Nominated	0.16	(.20)	.417	1.18 (0.79, 1.75)	0.16	(.20)	.417	1.18 (0.79, 1.75)	0.16	(.20)	.417	1.18 (0.79, 1.75)
r^2 for step	.18				.18				.18			
Block 2												
Neighbor Proximity	1.23	(.50)	.014	3.44 (1.29, 9.17)								
Group Proximity					0.99	(.46)	.033	2.70 (1.09, 6.70)				
Proximity Distance Change									-.24	(.14)	.080	0.78 (0.59, 1.03)
Total r^2	.21				.20				.20			
χ^2 (df) *p*	47.59 (8)		.001		46.37 (8)		.001		45.42 (8)		.001	

Notes. *N*=586 dyads (entire sample). Unstandardized beta weights and 95% confidence intervals for odds ratios are reported. Dyad gender: 1=same-gender, 2=other-gender. Dyad ethnicity: 1=same ethnicity, 2=different ethnicity. Teacher seating strategy: 1=no student input, 2=student input. Classroom layout: 1=tables, 2=rows. Neighbor proximity and group proximity: 1=moving apart (*n*=41 new non-neighbors; *n*=50 new non-groupmates) or staying apart (*n*=491 stable non-neighbors; *n*=466 stable non-groupmates), 2=getting closer (*n*=46 new neighbors; *n*=59 new groupmates) or staying close (*n*=8 stable neighbors;

n=11 stable groupmates).
